# Eumycetoma with pulmonary dissemination an unusual complication: Case series and literature review

**DOI:** 10.1371/journal.pntd.0010867

**Published:** 2022-11-10

**Authors:** Sarah Ali Yahya Adam, Eiman Siddig Ahmed, Salma Ibrahim Mohammed Adam, Omnia Babekir Abdallah, Emmanuel Edwar Siddig, Ahmed Hassan Fahal

**Affiliations:** Mycetoma Research Centre, University of Khartoum, Khartoum, Sudan; Yale University School of Medicine, UNITED STATES

## Case presentation

### Case No. 1

The patient was a 30-year-old jobless female from Sennar State, Central Sudan. In 2005, she stumbled during walking without obvious injury, which was followed by the appearance of a small painless swelling in the right big toe that rapidly increased in size. She consulted a traditional healer, and multiple skin incisions were done without improvement. Then, she consulted a local health center where an anti-tetanus toxoid injection was given, and her condition got worse, and she ended with a right big toe amputation 20 days later. Fifteen days following that, she noticed a painful swelling in her popliteal region that gradually increased in size. In 2008, multiple sinuses appeared on the swelling and started to discharge black grains. She was referred to the Mycetoma Research Center (MRC), Khartoum, for further management. She had a surgical biopsy that revealed evidence of *Madurella mycetomatis* eumycetoma, and she was started on 400 mg itraconazole daily and 5 mg folic acid daily. In 2009, she had wide local surgical excision of the mass. However, she stopped treatment as she assumed she was cured. The popliteal swelling recurred in 2012 at the surgical scar, and it rapidly progressed in size to affect the whole area and multiple sinuses discharging blood, and black grain appeared. She did not seek medical advice till 2015 when she was seen at the MRC.

In 2019, she developed a dry cough, not responding to medication. In February 2020, the cough became productive with blood that increased intensity and frequency. Her condition progressed, and in 2021, she developed central chest pain, which was dull, crashing in nature, and was associated with shortness of breath and palpitations. Three months later, she experienced lower right chest side sharp pain aggravated by coughing and movement more during the night, which was relieved by analgesics. She had daily low-grade pyrexia for more than 3 years, more aggravated at night. In March 2021, the cough became painful and forceful, containing black grains. The cough increased in frequency and interfered badly with her sleep. The coughed black grains increased in amount gradually. She developed a poor appetite, fatigability, headache, and joints pain and became depressed, which had a negative psychosocial impact on her and her family.

On clinical examination, the patient looked depressed, emaciated, and wasted. She was not pale, neither jaundiced nor cyanosed. Head and neck examination was within normal. The respiratory system examination showed normal chest skin, the right side was moving less, tactile vocal resonance was more on the right side, percussion note was dull on the right lower side, and there was decreased air entry at the right side but no added sounds. No abnormality was detected on the cardiovascular and central nervous systems or abdominal examinations.

**Fig 1:**
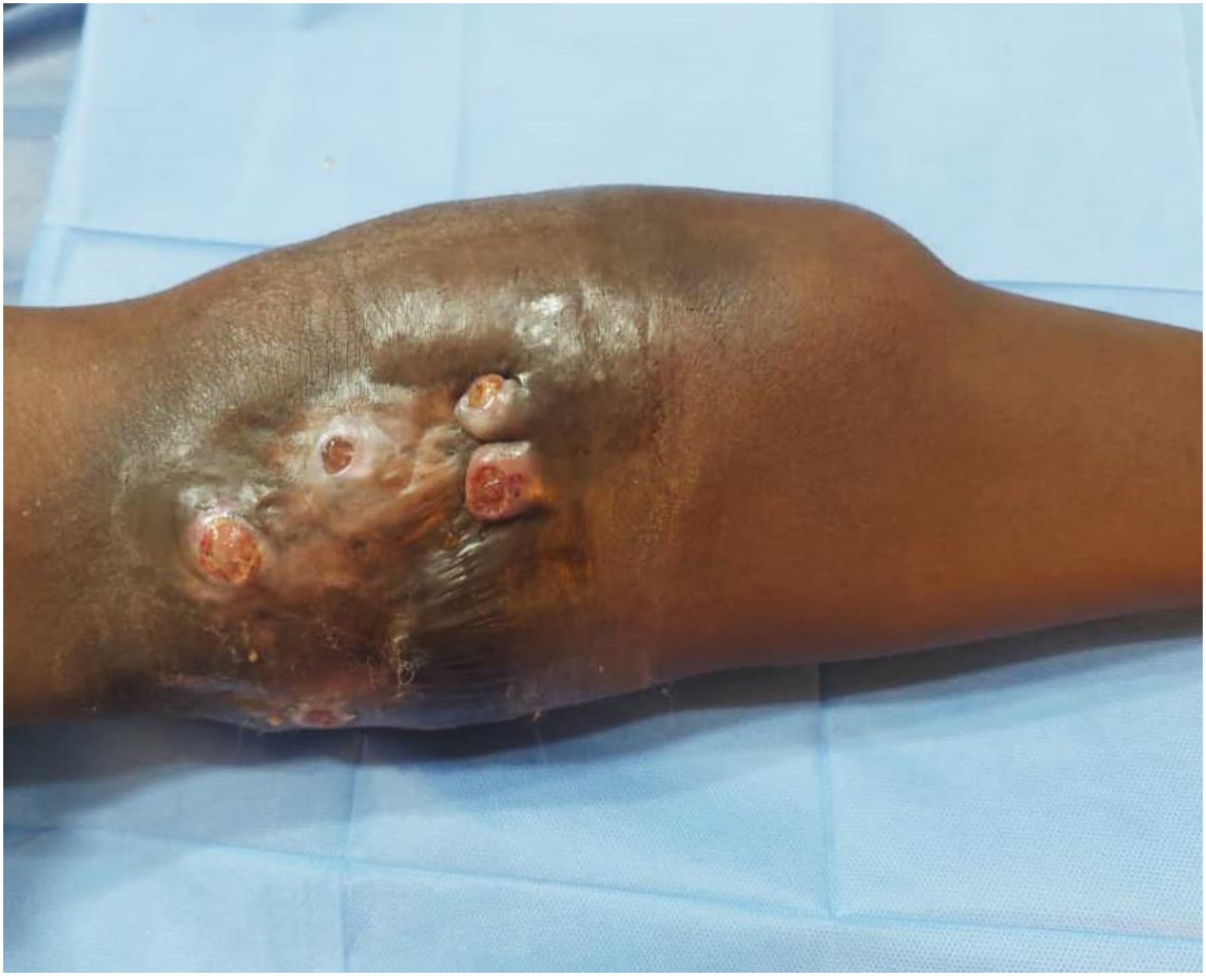
Showing massive swelling affecting the knee region, studded with multiple sinuses discharging purulent and seropurulent discharge containing black grains.

Local right knee joint examination revealed massive multiple nontender masses affected the whole region. They are studded with multiple sinuses discharging purulent and seropurulent discharge containing black grains. They were firm in consistency, attached to the skin and deep structures. The knee joint movements were restricted. The regional inguinal lymph nodes were enlarged. There were multiple enlarged veins above the popliteal area (Fig 1).

**Fig 2:**
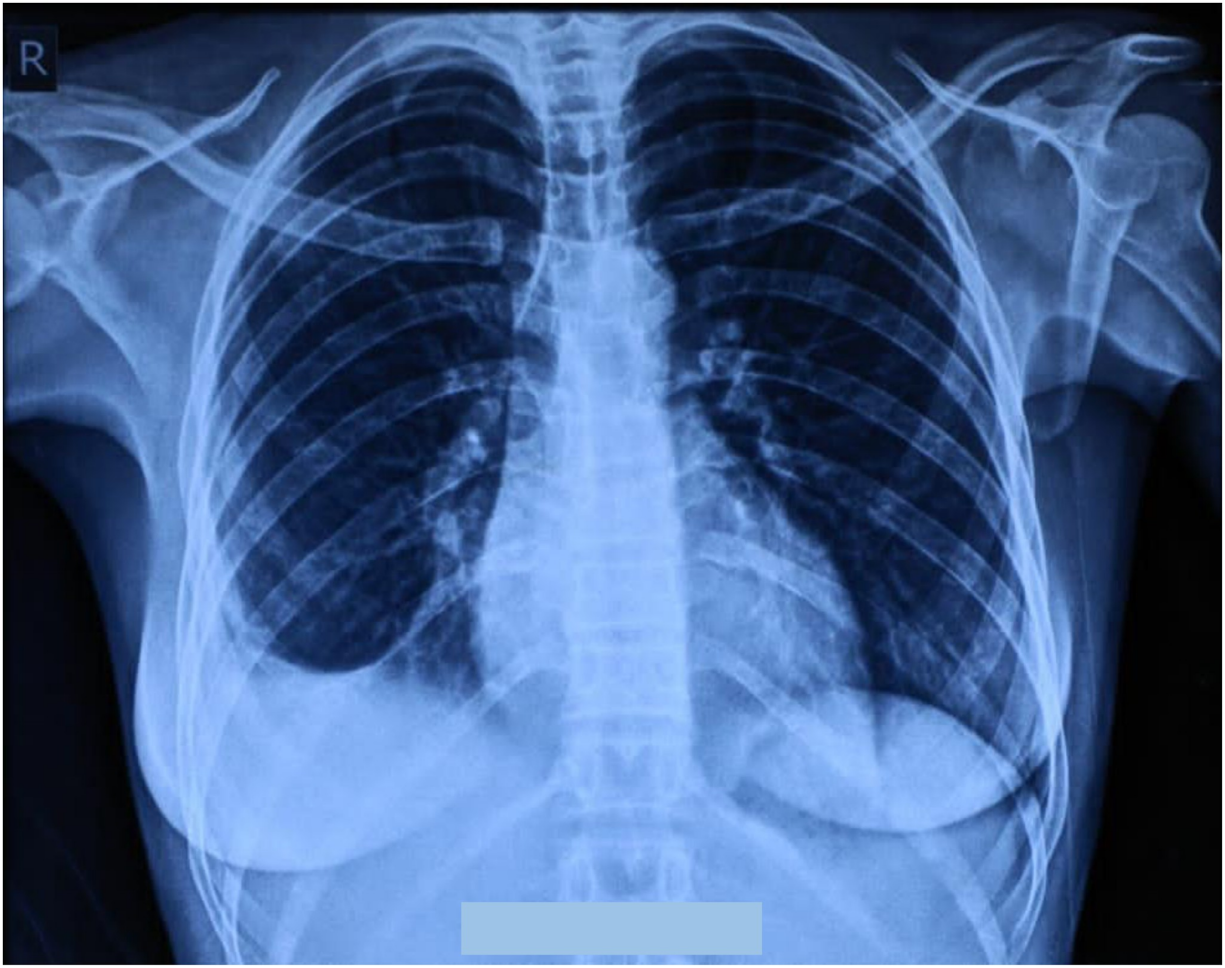
Chest X-ray revealing right pleural effusion and suggestion of basal pneumonia.

**Fig 3:**
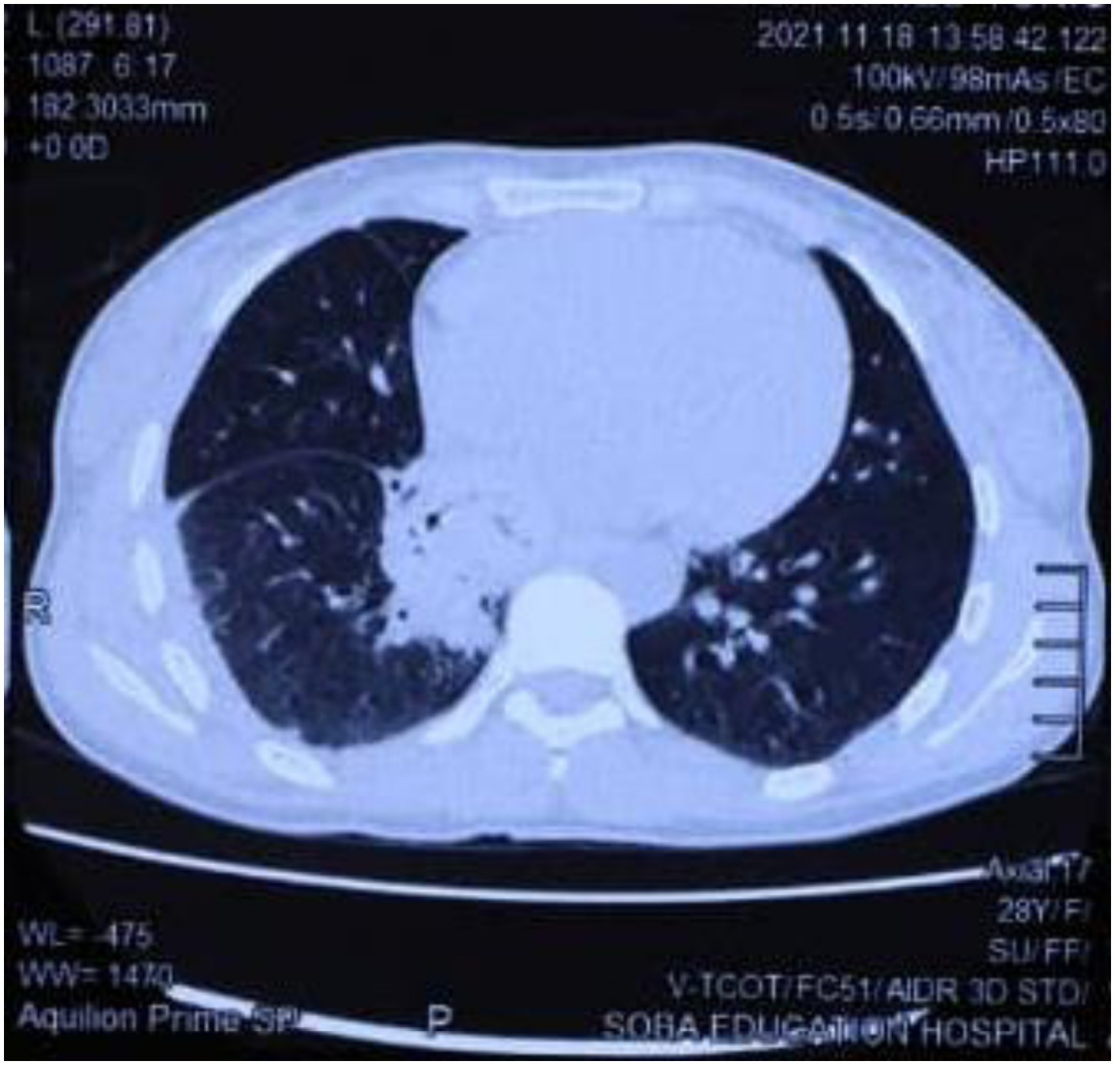
Chest CT showing segmental/lobar pneumonia with parapneumonic pleural effusion.

Her full blood count showed WBCS of 5.7 * 10^3^ micro/L, RBCs of 5.10 * 10^6^ micro/L, haemoglobin of 10.4 g/dl, MCV of 66.7 fL, MCH of 20.4, MCHC of 30.6 pg, platelets of 33910^3^ micro/L, neutrophils of 59%, lymphocytes of 31%, and monocytes of 8%. Her renal and hepatic functions tests were within normal. Furthermore, patient was tested for HIV and the result was negative. Chest X-ray revealed right pleural effusion and suggestion of basal pneumonia (Fig 2). Chest CT showed segmental/lobar pneumonia with parapneumonic pleural effusion (Fig 3). The grains obtained from the cough were positive for *M*. *mycetomatis* using species-specific primers as described previously [1].

She was on itraconazole 400 mg daily. During her last visit to the MRC, she was started on 1 g of ceftriaxone intravenously, twice daily for 10 days for her chest infection that had improved her respiratory symptoms, but she continued coughing the black grains. She suddenly developed right-sided hemiplegia, and a brain CT scan showed oedenomatous left cerebral hemisphere associated with pathological T2 and FLAIR hyperintensity involving the left frontal lobe, left parietal lobe, left occipital lobe, and left temporal lobe with involvement of left basal ganglia and corona radiate suggested meningoencephalitis. The patient died suddenly 3 days after this event.

### Case No. 2

The patient is a 32-year-old male from Kordofan, Sudan, who recently presented to the MRC coughing black grains for 2 weeks associated with recurrent attacks of mild fever, preceded by coughing blood once. The patient is known case of right foot eumycetoma since 2006.

**Fig 4:**
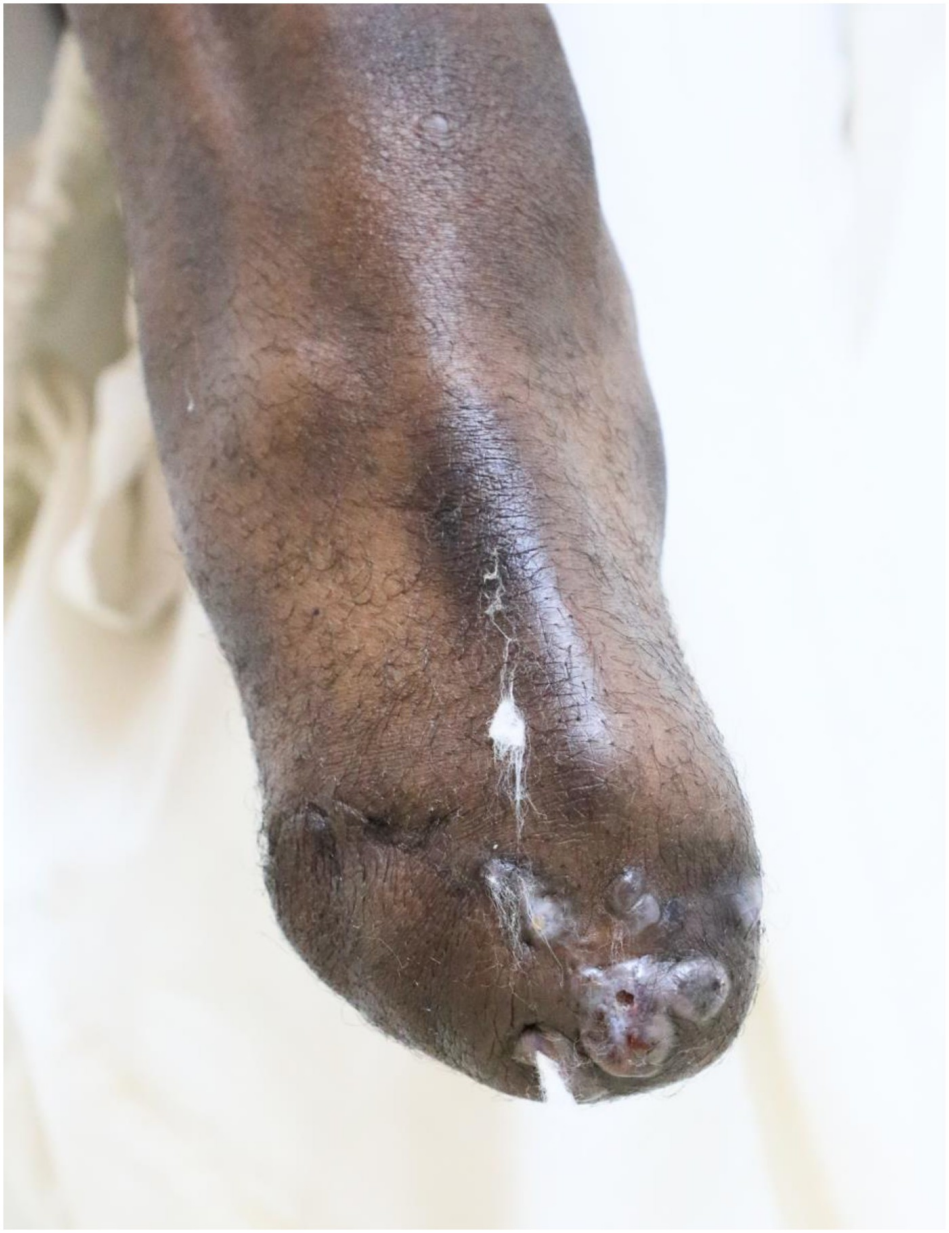
Photograph showing recurrence at the right below-knee stump.

**Fig 5:**
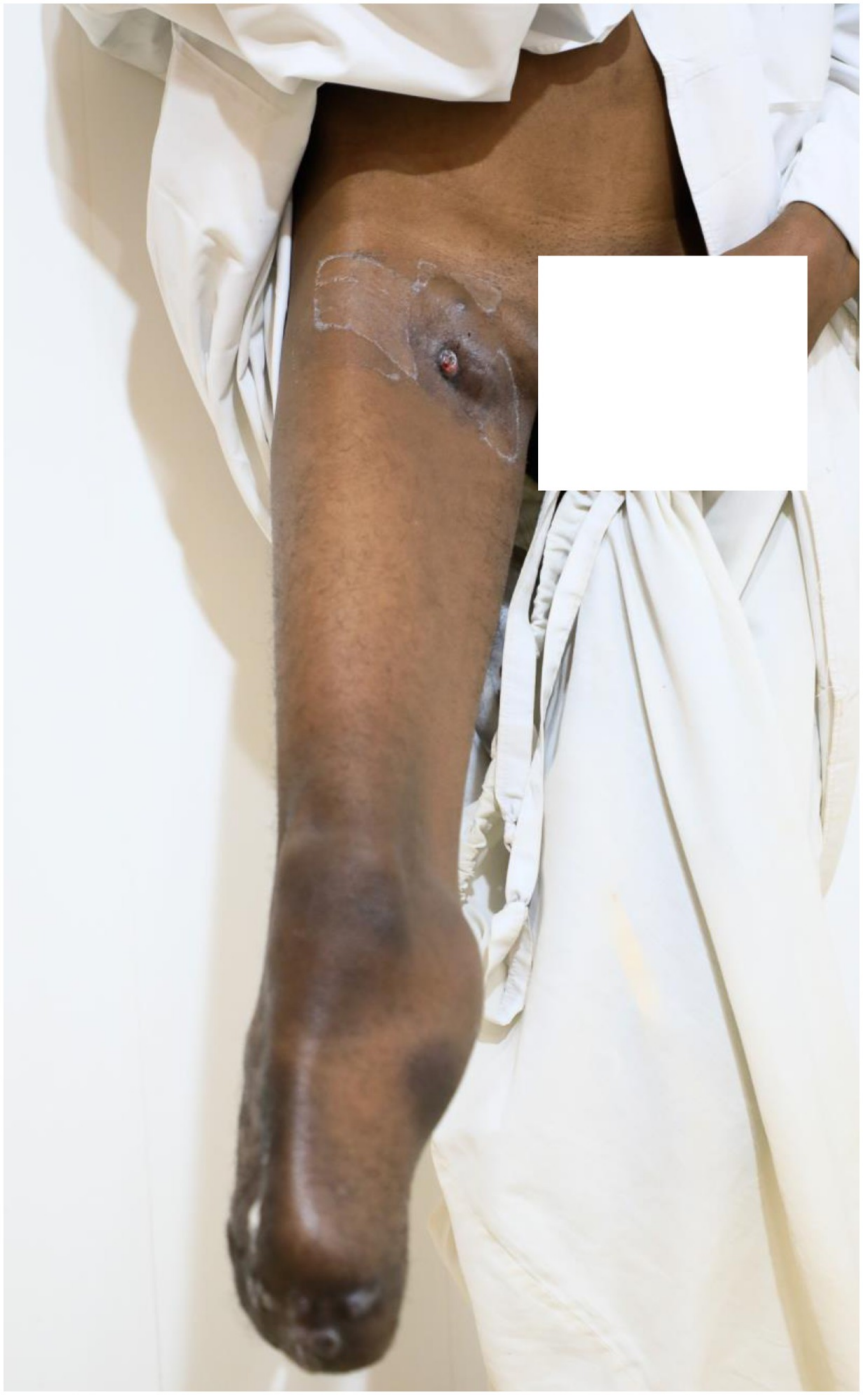
Photograph showing the secondary right inguinal mycetoma lesion.

His condition started 1.5 years prior to his presentation to the Mycetoma Research Centre, Soba University Hospital, in 2006. At presentation, he had a painless swelling in his right foot following a thorn prick. The swelling gradually increased in size, and then multiple sinuses discharging black grains appeared. That swelling was excised at a district clinic in his village. At the MRC, he presented with recurrent right foot swelling, 10 × 5 cm in diameter, with active and healed sinuses. Fine needle aspiration for cytology revealed eumycetoma infection caused by *M*. *mycetomatis*. He underwent wide surgical excision and was given Ketoconazole at a dose of 400 mg daily and recommended to attend outpatients clinic follow-up every 6 weeks. However, he was lost for follow-up. In 2017, he underwent a below-knee amputation of the affected limb for uncontrolled disease. In 2021, he started to develop signs of recurrence at the right stump (Fig 4). He then developed new masses in the right iliac fossa and inguinal region (Fig 5).

At a recent presentation to the MRC, on general examination, he looked unwell, pale, and vitally stable, with pulse rate of 72 beats/minute, respiratory rate of 20 breaths/minute, and blood pressure of 131/87 mm Hg. The head and neck examination was unremarkable. Chest examination showed no signs of respiratory distress, no chest deformity and the trachea was central. Both right and left sides moved equally. No palpable masses were detected. Chest percussion showed stony dullness on the right middle area of the lung and hyperresonance on the lower left side. There was decreased air entry on the lower field and more on the left lung. Mild crackles over the left side and decreased tactical fremitus on the lower part of both sides were detected.

The abdominal examination showed a small mass on the right iliac region, 3 × 2 cm in diameter, with no discharging sinuses and no tenderness noted. The rest of the examination was normal. A 5 × 5 cm in diameter mass that was not tender located in the right inguinal area was detected. It had a single active sinus discharging purulent fluid, black grains, and multiple healed sinuses. Local examination of the right below-knee stump showed a nontender swelling, 6 × 4 cm in diameter, with few nonactive sinuses. Other systems examinations were unremarkable.

Investigations showed low haemoglobin of 8.0 g/dl with normal leukocyte and differential count (neutrophils of 61%, lymphocytes of 29%, and monocytes of 7%) and normal platelet count. The renal profile showed blood urea of 11 mg/dl, low creatinine of 0.3 mg/dl with slightly low Na^+^ of 134 mmol/l, and K^+^ of 3.1 mmol/l. The liver function test showed elevated ALP of 222 U/l, but other enzymes were normal, AST and ALT were 17 U/l and 8 U/l, respectively. Total protein was within normal of 8.6 g/dl and low albumin of 2.7 g/dl. HIV test was negative for this patient.

**Fig 6:**
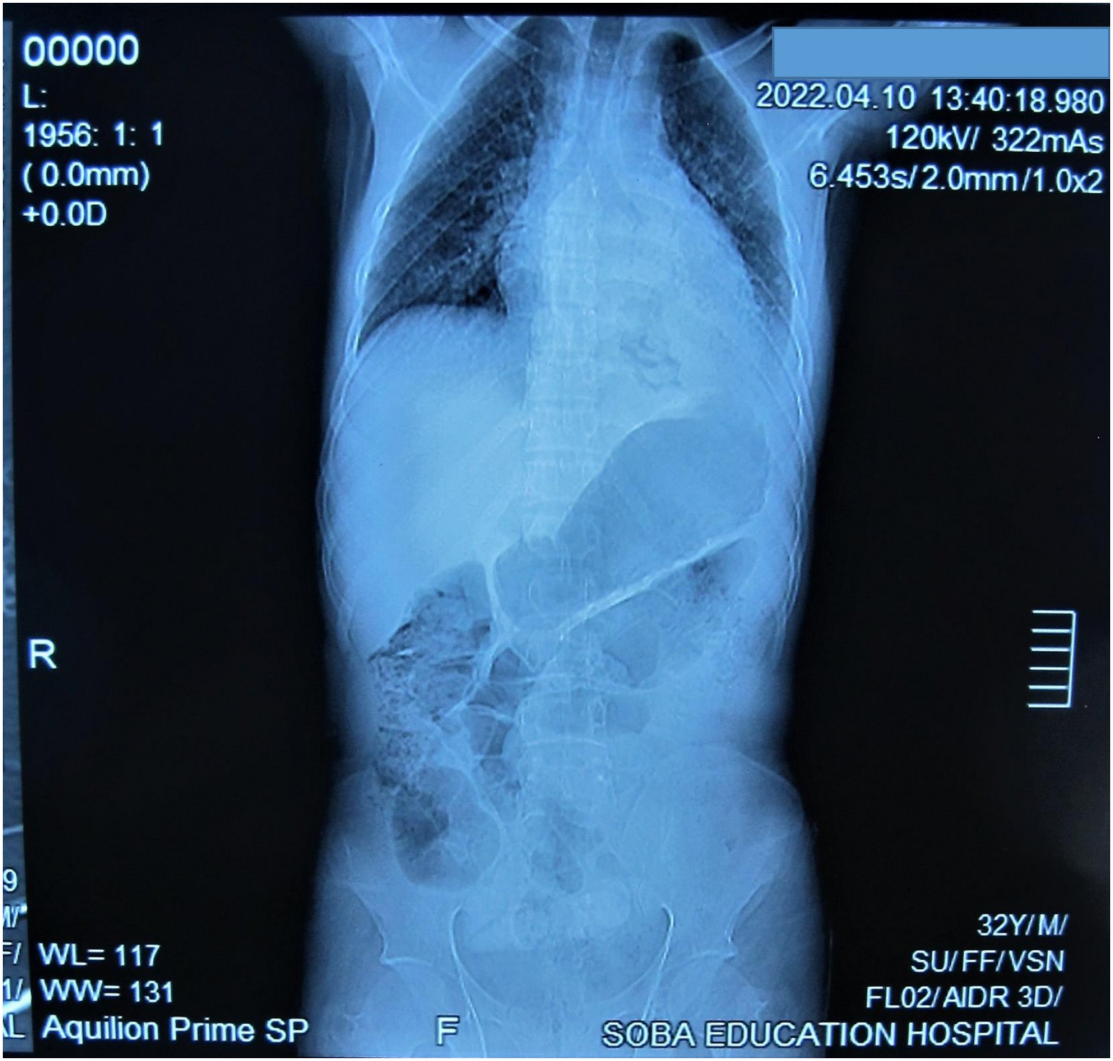
Chest CT and abdominal scan showing massive left lower lobe pulmonary consolidation and cavitary lesions.

He underwent a CT scan of both his chest and abdomen. CT chest revealed that the left lower lobe was completely replaced with consolidation and cavitary lesions containing small scattered grains, and the rest of the lung showed multiple small nodules with cavitary lesions with irregular outlines and thickness, while the right lung showed an ill-defined nodule at the anterior segment of the right lower lobe (Fig 6). CT abdomen revealed retrocrural extension of the left lung pathology reaching the upper border of the pancreas. There was a 44 × 219 mm right external iliac mass, mostly lymph node with peripheral enhancement. Another similar right superficial enhancing inguinal node was also seen.

An incisional biopsy of the right inguinal swelling revealed eumycetoma caused by *M*. *mycetomatis*, and the inguinal lymph nodes were reactively hyperplastic.

## Discussion

Mycetoma is one of the badly neglected tropical diseases reported worldwide but is endemic in many tropical and subtropical regions [2]. It is a chronic subcutaneous granulomatous inflammatory disease that spreads to affect the skin and deep structures, leading to massive tissue destruction, deformities, and disabilities. It can be fatal if not treated appropriately [3]. Mycetoma is caused by true fungi (eumycetoma) or certain actinomycetes (actinomycetoma) [4]. The triad of painless subcutaneous swellings, multiple sinuses, and seropurulent or purulent discharge that contain grains is pathognomonic of mycetoma [5]. Mycetoma is basically a localised disease and rarely can spread to the regional lymph node where secondary satellite lesions may develop. [6] Haematogenous spread is a rarity, reported in a few cases [7,8].

In this short communication, we report on 2 eumycetoma patients lung involvement from knee mycetoma, which is an uncommon presentation of this infection. Both patients were diagnosed as pulmonary mycetoma based on their clinical presentation, since both of them presented at the MRC coughing black grains. For the first case, we were able to accurately identify the pulmonary involvement by doing molecular diagnosis from the grains sample, which were found in the coughing materials obtained from the patient and turned to be eumycetoma due to *M*. *mycetomatis*.

A medical literature search revealed only 3 reports on the pulmonary mycetoma spread, and, interestingly, all of them were of knee eumycetoma origin [9,10]. Distant spread is not a frequent characteristic of eumycetoma, whereas, in actinomycetoma, that is common [11]. The explanation is not clear. Nevertheless, the absence of a capsule around the actinomycetoma lesion may facilitate the distal spread via the lymphatics and blood vessels.

The cause of distant spread in the reported patients is unclear. All of them were young and had no concomitant medical disease or obvious aggravating factors. However, the long disease duration and treatment interpretation were common features among the reported patients. All patients had knee lesions spread from foot lesions, and the explanation of this observation is an enigma. One of the explanation of pulmonary involvement maybe attributed to the invasion of the causative agent to the big vessels in the popliteal region, and then spreading haematogenously to the lung. Additional explantation may attributed to new inoculation of the causative agents into the thoracic wall during carrying vegetation on their backs.

Interestingly, one of the previously reported pulmonary disseminating eumycetoma patients had aggressive lower limb eumycetoma that was not controlled even by limb disarticulation; he had massive abdominal mycetoma spread, and the pulmonary spread was most probably by direct spread to the pleural cavity and the lung [6]. The massive surgical excisions of the knee eumycetoma in the reported patients may have led to further local or blood spread.

The cause of the aggressive clinical behaviour observed in the reported patients may be multifactorial. The causative organisms may of aggressive behaviour or do not respond to the few available antifungals. Hence, the causative organism identification to the species level and drug sensitivity are mandatory to know the possible treatment outcome in mycetoma. Drug resistance, especially with the interrupted treatment, may be another important cause of this reported aggressive behaviour.

All these patients had long disease duration and interpreted treatment courses, which can be due to their low health education and socioeconomic status. Thus, there is a great need for health education improvement, treatment adherence policy, early case detection, and management campaign; as for now, there is no control or prevention programme.

The treatment for mycetoma patients with pulmonary dissemination options is limited. Surgical excision option in such patients is not practical due to difficulty in radical surgical excision.

The only option for these patients is supportive symptomatic management. This poor outcome emphasises the importance of early case detection and management, good health education programmes, and pharmacy services for good medicine compliance.

**Key Learning Points**

- Mycetoma is a well-localised subcutaneous infection, yet rarely, lung spread is a scarce.
- Lung metastasis may be explained by a vascular or lymphatic spread of the causative organism from the primary site.
- In most cases, pulmonary disseminating eumycetoma has a poor prognosis and ends with the patient’s death.

**Top Five Papers**

1. Bakhiet SM, Fahal AH, Musa AM, Mohamed ESW, Omer RF, Ahmed ES, ET AL. A holistic approach to the mycetoma management. PLoS Negl Trop Dis. 2018 May 10;12(5):e0006391.
2. Siddig EE, El Had Bakhait O, El Nour Hussein Bahar M, Siddig Ahmed E, Bakhiet SM, Motasim Ali M, et al. Ultrasound-guided fine-needle aspiration cytology significantly improved mycetoma diagnosis. J Eur Acad Dermatol Venereol. 2022 Oct;36(10):1845–1850.
3. Siddig EE, Verbon A, Bakhiet S, Fahal AH, van de Sande WWJ. The developed molecular biological identification tools for mycetoma causative agents: An update. Acta Trop. 2022;225:106205.
4. Fahal AH, Suliman SH, Hay R. Mycetoma: The Spectrum of Clinical Presentation. Trop Med Infect Dis. 2018 Sep 4;3(3):97.
5. Siddig EE, Ahmed A, Ali Y, Bakhiet SM, Mohamed NS, Ahmed ES, Fahal AH. Eumycetoma Medical Treatment: Past, Current Practice, Latest Advances and Perspectives. Microbiology Research. 2021;12(4):899–906.

## Ethics statement

This study was approved by the Mycetoma Research Center review board (IRB2021/No.7). Written informed consents were obtained from the reported patients.
